# A Novel Way Of Repair Of Insulation Breaks During Pacemaker Generator Replacement

**Published:** 2009-09-01

**Authors:** Syed Manzoor Ali, Khurshid Iqbal, Nisar A Tramboo, Aijaz A Lone, Suresh Kaul, Neelam Kaul, Imran Hafiz

**Affiliations:** Department of Cardiology Sheri Kashmir Institute of Medical Sciences, Soura Srinagar 190010

**Keywords:** Pacemaker lead, repair of insulation breaks

## Abstract

Minor abrasions  can occur while mobilising old lead during pacemaker generator replacement necesittating placement of additional lead adding to the financial burden and junk in heart. We describe a novel way of repair of old pacemaker lead preventing additional lead placement.

## Introduction

 Minor abrasions of the pacemaker lead can occur during manipulation of old lead at the time of   generator replacement necessitating replacement of whole system adding to the complexity of the procedure and financial burden to the patient. We describe a novel way of sealing the insulation break preventing lead replacement.

## Procedure

**Figure 1:**
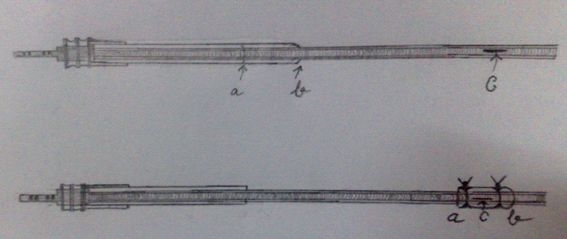
Diagram showing a circumferential incision given at "a" in the outer polyurethane jacket and mobilizing the segment "a b" to the rent in the insulation at "c".

A 65 year male with history of complete heart block on single chamber pacemaker of 11 year duration was admitted with capture loss and analysis indicated depleted battery with fall of magnet rate. Patient was taken for generator replacement and while removing the old generator and mobilizing the lead there was a small abrasion in the insulation of the lead  resulting in seepage of blood into the lead  and analysis showed fall of lead impedance to 200 ohm, minimal pacing threshold of 2.5V, 0.4 ms and R wave of 4mV compared to last recorded parameters, impedance 830 ohm, pacing threshold 0.7V, 0.4ms and R wave of 15mV. Since patient was poor and could not afford the cost of lead an attempt was made to repair the old lead by mobilizing a one cm sleeve of insulation material carefully separated by  an encircling incision in the outer  polyurethane  jacket present on the lead on the connector side. The sleeve was carefully advanced over to the rent in the insulation and tied on the two sides firmly by 2-0 Prolene thereby completely sealing off the rent and the repeat analysis showed normal lead impedance of 800 to 850 ohm, minimal pacing threshold of 0.7V, 0.4ms and R wave of 15mV (Figure 1). Pulse generator was closed and patient discharged and is on follow up with normal parameters.

## Discussion

In our territory conduction abnormalities are quite a problem with an average of 1-2 permanent pacemaker implantations a day and our centre has an experience of more than 4000 implantations involving single chamber, dual chamber, biventricular pacemakers, ICDs and CRTD devices.  In the past various biocompatible materials like medical adhesives, commercially available patch kit or silicone film have been used to seal off the insulation breaks in the pacemaker leads [1,2]. However no technique has been authenticated keeping in view the proper sterilization of the material and rejection of the material by body tissues and also the availability of such materials at the time of such relatively rare happenings. Most of the operators tend to place new leads. Although relatively easier, it adds to the financial burden and places additional lead in the human heart as it is not possible to remove the old lead easily and may need laser excimer sheaths [3] for extraction. Hence sealing the insulation using the material of the same lead helps many ways. It avoids use of materials about which body compatibility is not known. Since it does not need to be sterilized hence can be used any time and avoids any chance of infection. There are no additional leads needed in the heart and since the lead is already fixed patient can be discharged early  without any risk or lead displacement or rise in threshold. However the procedure needs great care to separate a sleeve of insulation material and transfer it to the location without any longitudinal incision in it.
